# Atypical granular cell tumour in a child: A rare case report

**DOI:** 10.1002/ski2.218

**Published:** 2023-03-10

**Authors:** Yuma Kitahara, Catherine Elizabeth Hook, Kana Miyagi, Nigel P. Burrows

**Affiliations:** ^1^ Department of Dermatology Addenbrooke's Hospital Cambridge University Hospitals NHS Foundation Trust Cambridge UK; ^2^ Departments of Pathology Addenbrooke's Hospital Cambridge University Hospitals NHS Foundation Trust Cambridge UK; ^3^ Departments of Plastic Surgery Addenbrooke's Hospital Cambridge University Hospitals NHS Foundation Trust Cambridge UK

## Abstract

We report an atypical granular cell tumour (GCT) presenting in a 6‐year‐old boy. GCTs are of neural origin and most cases arise in patients between the ages of 40 and 60. There are few reported cases in children, in whom malignant presentations are exceptionally rare. This patient presented with a 1 year history of a slowly enlarging nodule on the right anterior abdomen. Examination revealed a firm, nodular dermal skin lesion, which was fully excised. Histology revealed an atypical GCT.

## Graphical abstract

We report a rare case of atypical granular cell tumour presenting in a 6‐year‐old boy. We report the presentation, excision, and histological findings of this unique case.

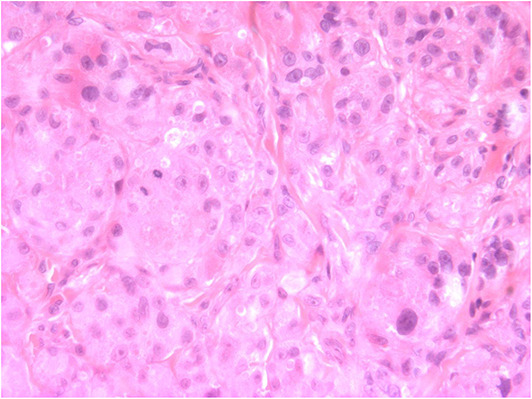

## 1 INTRODUCTION

Granular cell tumours (GCT) are characterised by eosinophilic cytoplasmic inclusions of peripheral nerve myelin proteins.^1^ The histogenetic link between Schwann cells and granular cell tumours has been established and the tumour is believed to have a neural origin despite its original description as a myoblastoma.^2^, ^3^ Most cases of GCT develop in patients between the ages of 40 and 60 years and it is rare in the paediatric population. We report the presentation of an atypical GCT in a 6‐year‐old boy.

## 2 CASE REPORT

**FIGURE 1:**
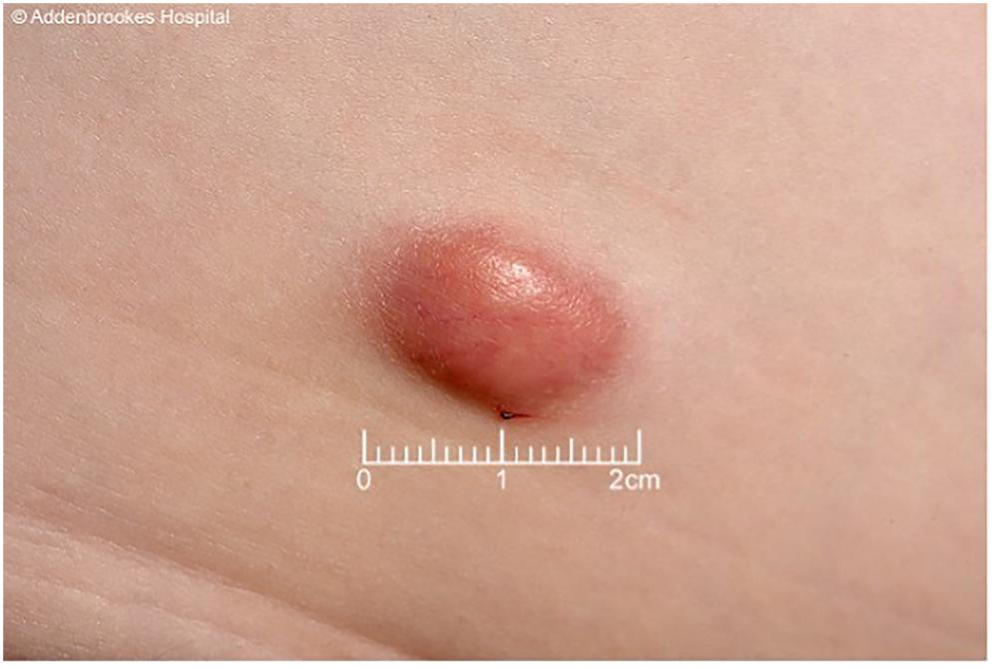
Firm 2 cm dermal nodule with erythematous margin, located over the right lower abdomen

A 6‐year‐old boy presented with a 1‐year history of a slowly enlarging, asymptomatic nodule over the right anterior abdomen. Examination revealed a firm, nodular, dermal skin lesion measuring 20x11 mm. The skin overlying was erythematous (Figure 1).

**FIGURE 2:**
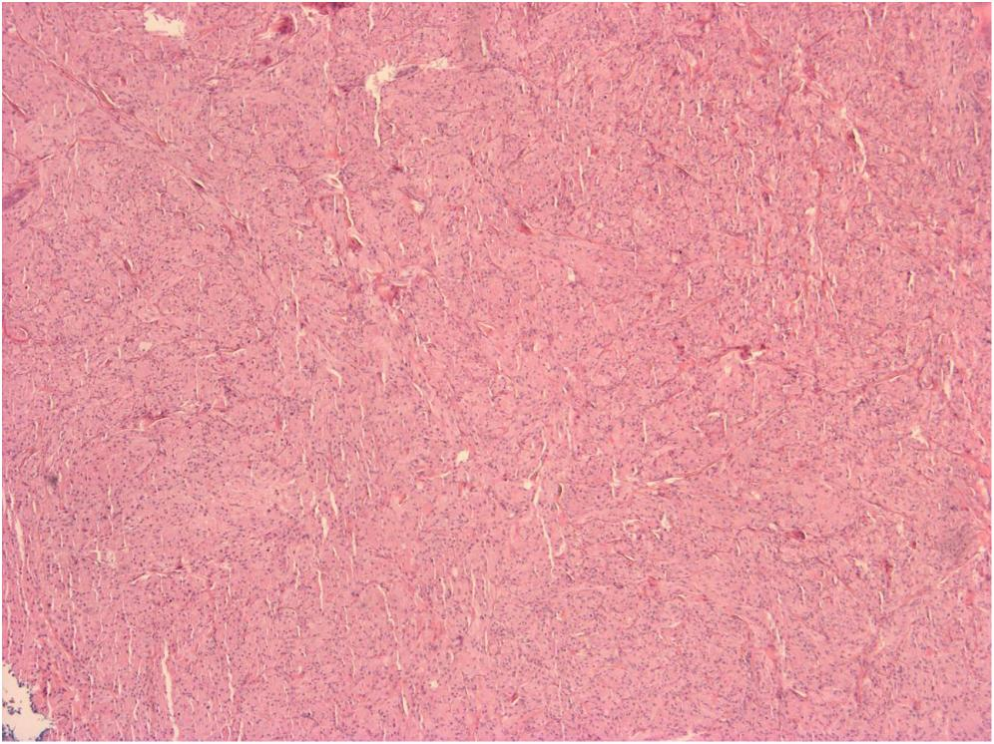
Low power (x40) diffuse view of the lesion showing relative uniformity

**FIGURE 3:**
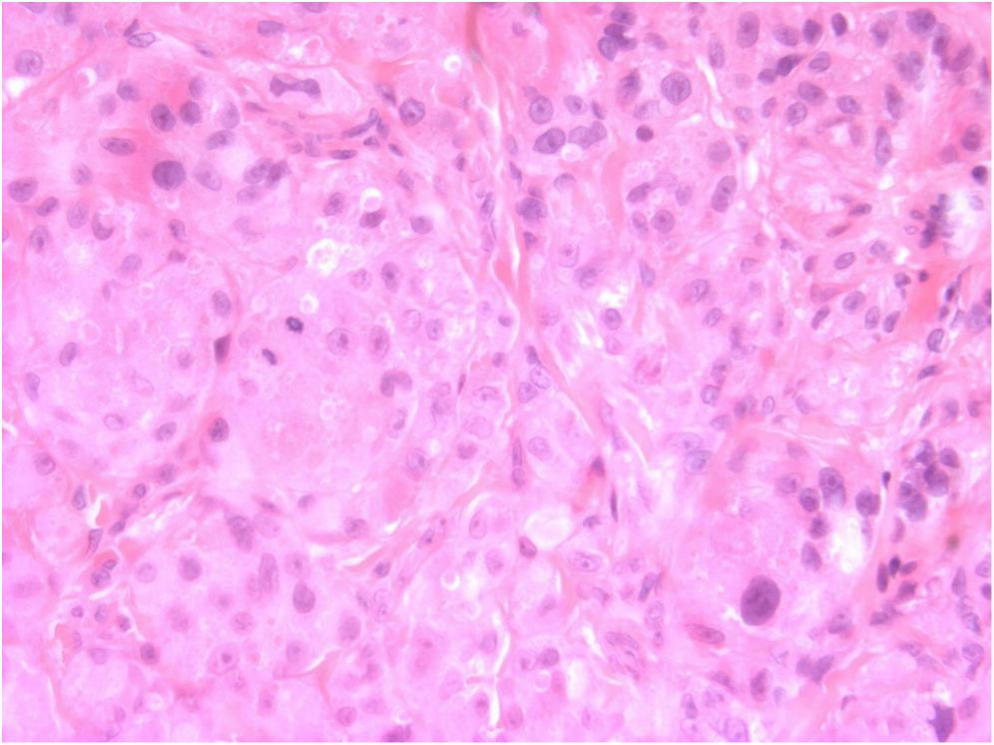
Power field (x400) shows rounded to polygonal eosinophilic cells with abundant cytoplasm and prominent eosionophilic cytoplasmic inclusions, arranged in nests. There are two mitoses and variable nuclear size. The lesional cells were strongly S100 positive

The initial differential diagnoses included juvenile xanthogranuloma and dermatofibrosarcoma protuberans due to the erythematous, nodular, and subcutaneous appearance. A punch biopsy showed tissue infiltrated by large pale cells with small regular nuclei and “foamy” amphophilic cytoplasm. There were 5 mitoses per 10 high power fields (x200), suggestive of atypical GCT. The nodule was fully excised with a 3 mm margin, including a cuff of Scarpa's fascia to which the lesion had adhered. Subsequent histology confirmed a well‐circumscribed but non‐encapsulated dermal tumour. The morphological impression of GCT was supported through strong S‐100 staining on immunohistochemistry. There were 7 mitoses per 10 high power fields (x200) and the mib proliferation index was approximately 5% (Figures 2 and 3). The postoperative period was uneventful and there has been no local or regional lymph node recurrence 18 months following excision.

## 3 DISCUSSION

The typical presentation of a GCT is of a singular, asymptomatic, slow‐growing mass, most commonly seen in the skin, subcutaneous tissue or oral cavity.^4^, ^5^ Few GCTs are malignant with only 157 cases identified in a recent literature review.^6^ Paediatric presentations are rare, and of those reported only 3 were malignant.^7^, ^8^, ^9^ This case report adds to the existing literature with an unusual paediatric presentation of an atypical GCT.^10^

Dermal presentations of GCT are often non‐specific, but usually arise as solitary nodules. Multiple subcutaneous nodules should instead raise clinical suspicion for genetic conditions such as neurofibromatosis and Noonan's syndrome.^11^ The most common location of GCTs is the upper extremity.^12^ Presentation on the abdominal wall are rare and documented in only 29 reports.^13^

The classification of GCTs into benign, atypical, and malignant tumours is based on histological criteria and immunohistochemistry for S‐100 and CD68. Malignant GCTs exhibit three or more of the following features; necrosis, spindling of tumour cells, vesicular nuclei with large nucleoli, mitotic rate (greater than two mitoses/10 high‐power fields), high nuclear to cytoplasmic ratio, and pleomorphism.^12^ As in our case, a diagnosis of atypical GCT is only considered if one or two of these features are present. This lesion did not score sufficient features to be classified as malignant. Poorer survival is linked to the presence of local recurrence, metastasis, larger tumour size, older patient age, histologic classification as malignant, presence of necrosis, increased mitotic activity, spindling of tumour cells, vesicular nuclei with large nucleoli, and a proliferation index (such as Ki67 or Mib‐1) greater than 10%.^14^

The tumour extended deep to the scarpa's fascia, illustrating these tumours can behave in an invasive manner even if otherwise categorised as benign. Wide local excision and long‐term monitoring is recommended, as local recurrence is 2%–18%.^5^, ^12^

This case report highlights the clinical features of an unusual paediatric presentation of an atypical GCT. Granular cell tumour should be amongst the differential for solitary subcutaneous nodule and this case demonstrates the importance of histological sampling. There is need for follow‐up guidelines for GCT.^13^ We recommend 6 months follow‐up and subsequent yearly review as atypical granular cell tumours rarely may progress and metastasise.^15^, ^16^

## CONFLICT OF INTEREST

All authors declare no conflicts of interest.

## AUTHOR CONTRIBUTION

**Yuma Kitahara**: Project administration (Equal); Writing – original draft (Lead); Writing – review & editing (Equal). **Catherine Elizabeth Hook**: Writing – review & editing (Equal). **Kana Miyagi**: Writing – review & editing (Equal). **Nigel P. Burrows**: Conceptualization (Lead); Project administration (Equal); Writing – review & editing (Equal).

## ETHICS STATEMENT

Not applicable.

## Data Availability

The data that support the findings of this study are available from the corresponding author upon reasonable request.
